# Structure of prolylrapamycin: confirmation through a revised and detailed NMR assignment study

**DOI:** 10.1038/s41429-024-00714-6

**Published:** 2024-03-19

**Authors:** Annalisa Mortoni, Eugenio Castelli, Teresa Recca, Paolo Quadrelli

**Affiliations:** 1 Curia Italy S.r.l., Via Volturno 43, 20089 Rozzano, MI Italy; 2 Centro Grandi Strumenti (CGS), Università degli Studi di Pavia, Via Bassi 21, 27100 Pavia, Italy; 3 Dipartimento di Chimica, Università degli Studi di Pavia, Viale Taramelli 12, 27100 Pavia, Italy

**Keywords:** Structure elucidation, Drug discovery and development, Drug regulation

## Abstract

A complete and detailed characterization of Rapamycin (**1**) and Prolylrapamycin (**2**) has been conducted by homo- and hetero-nuclear NMR experiments in DMSO-*d*_6_ along with HRMS and FT-IR spectra and DSCs analyses. The NMR experiments allowed the assignment of every single proton and carbon atom belonging to the two structures and the definitive confirm of the presence of a pyrrolidine ring in Prolylrapamycin (**2**) in place of the piperidine ring that characterizes the structure of Sirolimus.

## Introduction

**Fig. 1:**
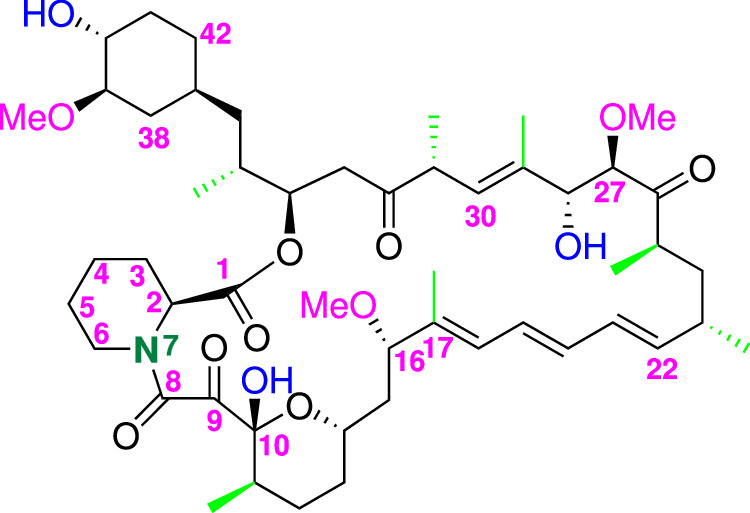
Chemical structure of Rapamycin **1** (C_51_H_79_NO_13_) with numbering

Rapamycin **1** (Sirolimus) (Fig. 1) is a well-known immunosuppressive metabolite produced from several actinomycete species [1]. The importance of compound **1** also relays in some additional therapeutic potentials such as antifungal, antitumor and neuroprotective and/or neurodegenerative activities.

A number of reviews and papers have been dedicated to the properties of these molecules as well as the perspectives in the use of such macrocycles in targeting specific proteins, aiming the isolation of new and more potent inhibitor of critical diseases [2]. The structure of Rapamycin **1** is, in fact, inserted among the inhibitors of mammalian targets (mTOR); these inhibitors have been applied in cancer research and in the field of inflammation, central nervous system diseases and antiviral infections [3].

Being a natural product isolated typically from *Streptomyces rapamycinicus*, compound **1** is accompanied by many derivatives whose identification deserves a great interest from two main reasons [4]; first, the possibility to dig up new and more potent inhibitors and antagonists in the diseases where Rapamycin finds application or even others; second, the characterization of impurities is required by regulatory institutions for the commercialization of APIs. In this view the identification of novel derivatives is reported in a number of papers in chemical literature where the structure of Rapamycin has been also revised in the light of modern NMR techniques [5, 6].

**Fig. 2:**
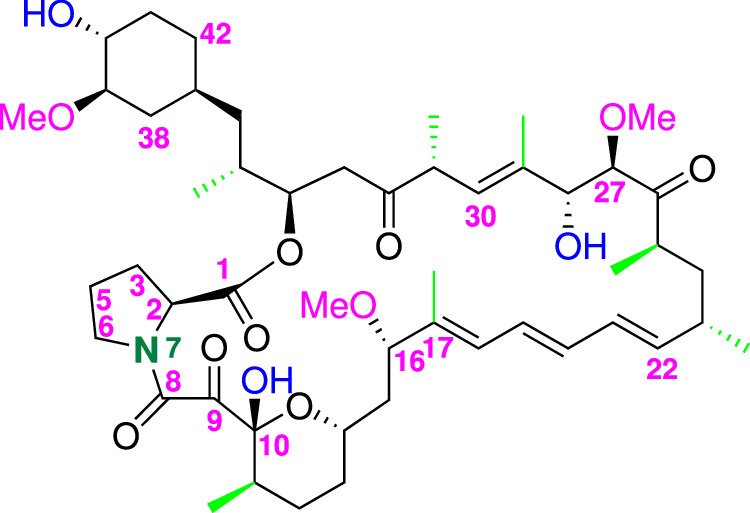
Chemical structure of Prolylrapamycin **2** (C_50_H_77_NO_13_) with numbering. Carbon 4 in the pyrrolidine ring is missing and numbering has been left identical to that of Rapamycin **1** in Fig. 3 for sake of comparison

The preparation, isolation and correct identification of new Rapamycin structure derivatives by microbial manipulations remains a topic of great interest for chemical companies that produce drugs and precursors of new active ingredients with specific pharmaceutical activities [7]. Rapamycin **1** is produced by fermentation of *Streptomyces hygroscopicus* bacterium in Curia Italy S.r.l. and a complete study has been conducted on the impurities profile contained in the final drug. One of these is known as Prolylrapamycin **2** (Fig. 2); from a literature survey, Prolylrapamycin is mentioned in few papers and its presence is related to the panel of impurities to be identified in the chromatographic analyses. Some indications relative to its structure are reported but not with a complete and detailed characterization.

In fact Prolylrapamycin **2** is cited to be structurally similar to Rapamycin **1** with the main change regarding the presence of a pyrrolidine ring in place of the piperidine that characterizes the Sirolimus structure (compare structures in Figs. 1 and 2).

However, in literature the structure of Prolylrapamycin **2** is reported, as we mentioned, with great emphasis with respect to the analytical determinations from the fermentation broth but with few details regarding a complete spectroscopic characterization and assignments in particular from NMR studies [7]. The Prolylrapamycin structure is considered attributed from the data reported in patents [8] or in other documents considered to be relevant where precise chemical data are often missing.

On pursuing its research on Rapamycin, Curia Italy S.r.l. and the Chemical Department of the University of Pavia joined their efforts in revising the chemical structure of Rapamycin **1** to be used as reference for a full characterization of a sample of Prolylrapamycin **2** in order to determine its chemical structure through a complete spectroscopic and physical chemical pattern of identification. This paper describes the isolation and comprehensive spectral characterization of Prolylrapamycin **2** using HPLC, HRMS, as well as one- and two-dimensional NMR techniques.

## Results

### Rapamycin **1**

A sample (6.6 mg) of Rapamycin (**1**) has been dissolved in DMSO-*d*_6_ and the ^1^H, ^13^C and ^15^N NMR spectra were recorded along with COSY, HSQC, HMBC, TOCSY and NOESY experiments at 700 MHz instrument (see experimental section and Supplemental Material for details).

Table 1 collects the ^1^H, ^13^C and ^15^N NMR chemical shift values that allowed to give the complete and updated assignments to all the proton and carbon atoms of compound **1** structure with, in addition, the chemical shift of the ^15^N atom belonging to the piperidine ring.

**Table 1:**
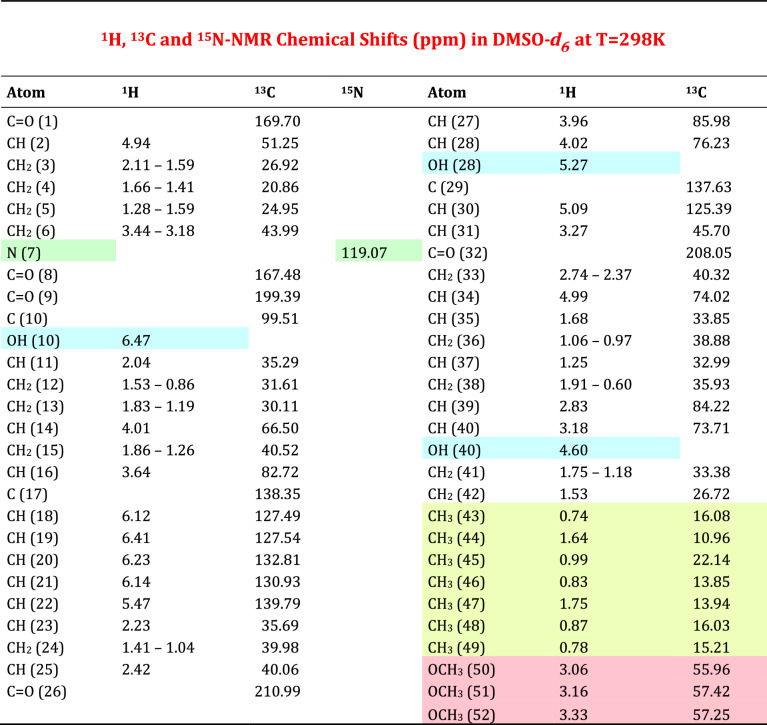
Compound **1** NMR assignments

The results obtained are in full agreement with those reported in the literature describing the structure of Rapamycin [6, 7]. The chemical shift values nicely support the reported structure of compound **1** and some key points can be highlighted (Fig. 1). 2D TOCSY experiment allowed to give the assignments to all ^1^H spin systems, in particular to those belonging to the piperidine ring, starting from proton atom (2) to proton (6); H(2) and (6) are bonded to the nitrogen atom (7) whose chemical shift nicely fit with the values reported for this type of heterocyclic compounds in literature [9].

The olefinic moiety of the molecule starts from the quaternary carbon (17) originating an hexatriene chain; the protons are found in the expected region from *δ* 5.5 to 6.4 ppm. Another olefinic proton is also found at carbon (30) at *δ* 5.09 ppm. All the methyls groups were detected and are reported in the last part of Table 1 (highlighted in light green) while the three methoxy groups were found in the range *δ* 3.1–3.3 ppm (see Table 1). All the other carbons and protons of the *sp*^3^ structure were also correctly assigned and listed in Table 1.

These findings were needed for the structural study we conducted on the impurity Prolylrapamycin (**2**) we discuss here below.

### Prolylrapamycin **2**

A sample (3.2 mg) of Prolylrapamycin (**2**) has been dissolved in DMSO-*d*_6_ and the ^1^H, ^13^C and ^15^N NMR spectra were recorded along with COSY, HSQC, HMBC, TOCSY and NOESY experiments at 700 MHz instrument (see experimental section and Supplemental Material for details). Table 2 collects the ^1^H, ^13^C and ^15^N NMR chemical shift values that allowed to give the complete and updated assignments to all the proton and carbon atoms of compound **2** structure with, in addition, the chemical shift of the ^15^N atom belonging to the pyrrolidine ring.

**Table 2:**
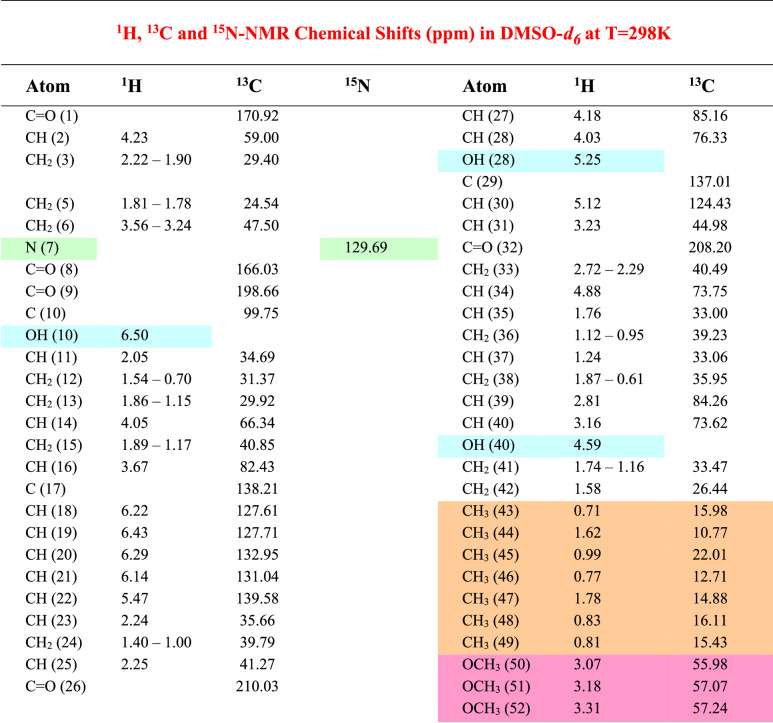
Compound **2** NMR assignments

**Fig. 3:**
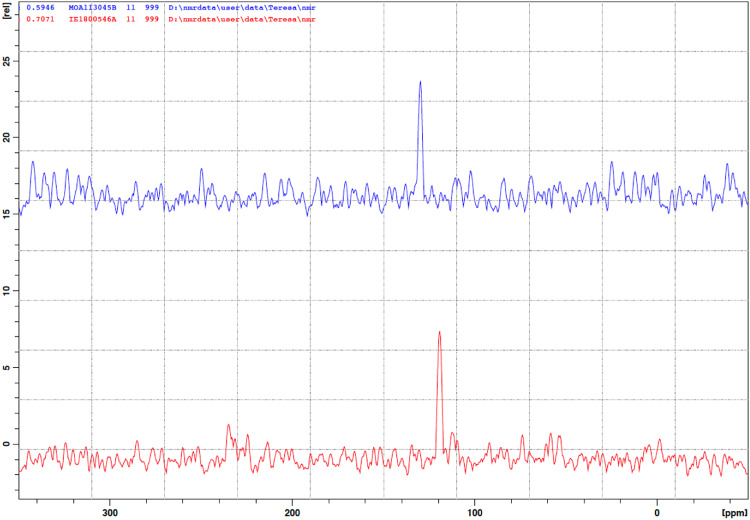
Comparison between ^15^N chemical shifts of Rapamycin (**1, red line**) and Prolylrapamycin (**2, blue line**)

The results obtained demonstrate that the structure of compound **2** differs from that of Rapamycin (**1**) for a single structural moiety, *i.e*. the presence of the pyrrolidine ring in place of the piperidine and these findings are in agreement with the suggestions reported in the literature describing the structures of Rapamycin impurities and side compounds derived from the fermentation processes [7]. Fig. 2 reports the structure assigned nicely supported by the NMR experiments that allowed to attribute all the correct chemical shift at proton and carbon atoms. In particular, the diastereotopic protons belonging to the pyrrolidine ring starting from carbon atom (2) to carbon atom (6) were correctly defined. For sake of comparison with the structure of compound **1**, numbering of the nitrogen-containing ring was left unchanged with deletion of the carbon (4) in compound **2**. Carbons (2) and (6) are bonded to the nitrogen atom (7) whose chemical shift nicely fit with the values reported for this type of heterocyclic compounds in literature [9]. We acquired ^1^H-^15^N HMBC experiment on compound **2** to assign the signal of N(7) atom of the pyrrolidine ring; Fig. 3 reports the comparison between the columns extracted by the 2D HMBC experiment, where the N chemical shift of Rapamycin (**1**) is found at *δ* 119.1 ppm while that of Prolylrapamycin (**2**) is slightly shifted downfield at *δ* 129.7 ppm (reference NH_3_ for the scale of *δ* = 0.0).

**Fig. 4:**
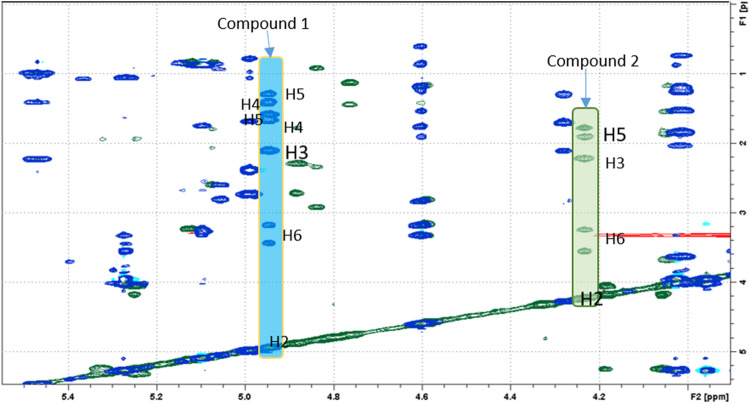
Overlapping of TOCSY experiments of compounds **1** (blue) and **2** (green) with highlight of the patterns of the rings. In compound **2** protons H4 are missing

Furthermore, through TOCSY experiments (Fig. 4), the comparison between the peaks relative to proton and carbon atoms relative to the nitrogen-containing rings clearly shows the absence of a CH_2_ (numbered as carbon 4) in the sequence starting from H2 proton.

The chemical shift of H2 is very different and in accordance with those of a piperidinic (referred to compound **1**) and pyrrolidinic (referred to compound **2**) ring. In fact, the pattern of the pyrrolidinic ring is very similar to that of a proline [9].

Concerning the general structure of Prolylrapamycin, we can observe that the olefinic moiety of the molecule starts from the quaternary carbon (17) originating an hexatriene chain; the protons are found in the expected region from *δ* 5.4 to 6.4 ppm. Another olefinic proton is also found at carbon (30) at *δ* 5.09 ppm. All the methyls groups were detected and are reported in the last part of Table 1 (highlighted in light yellow) while the three methoxy groups were found in the range *δ* 3.0–3.3 ppm (see Table 1). All the other carbon and protons of the *sp*^3^ structure were also correctly assigned and listed in Table 2.

**Fig. 5:**
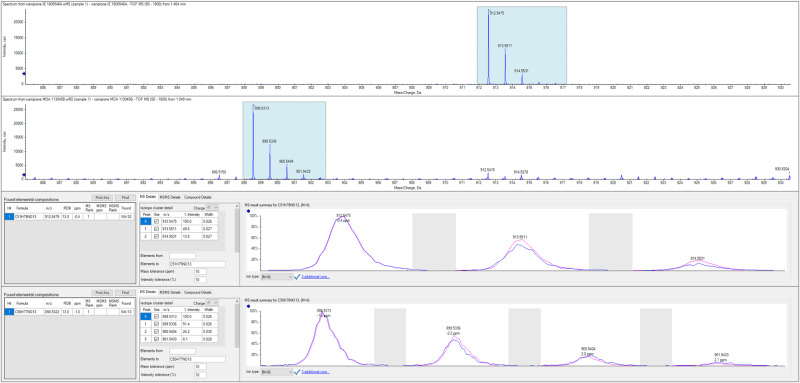
HRMS spectra of compounds **1** (top) and **2** (bottom)

The characterizations of both compounds **1** and **2** were completed with HRMS spectra (Fig. 5) that confirmed the lack of a CH_2_ in the mass of compound **2**, testifying the presence of the pyrrolidine ring as well as by FT-IR spectra. DSC analyses also revealed the decomposition of compound **1** just after the melting point (mp 189.7 °C) and the presence of a large melting band centered at 161.1 °C indicating that the compound contains impurities; also in this case a change of colour indicates decomposition after melting.

## Discussion and conclusions

We have reported the results of homo- and hetero-nuclear NMR experiments on Rapamycin (**1**) and Prolylrapamycin (**2**) in DMSO-*d*_*6*__DMSO-*d*6_ as well as HRMS and FT-IR spectra and DSCs analyses leading to a complete and detailed characterization of both the compounds. In particular the NMR experiments conducted allowed the assignment of every single proton and carbon atom belonging to the two structures and the definitive confirm of the presence of a pyrrolidine ring in Prolylrapamycin (**2**) in place of the piperidine ring that characterizes the structure of Sirolimus **1**. All the data reported in this work are consistent and coherent with the structures reported; specifically Prolylrapamycin (**2**) receives for the first time a detailed characterization beyond any reasonable doubt.

The structural modification occurring in compound **2** where the piperidine is replaced by the pyrrolidine ring causes a remarkable variation both in chemical shifts and proton couplings in the spectral attributions concerning those compounds where five-membered rings are found, as also reported in specific investigations from NMR literature [10]. In fact, ^1^H NMR spectra of compounds containing a piperidine ring as structural element, a characteristic subspectrum can be recognized as a result from a chair conformation of the nitrogen-containing six-membered ring. In compound **1**, although diastereotopic, five well-separated signals can be found in the range from *δ* 1.28 to 4.94 ppm (see Table 1). In contrast, the ^1^H NMR spectrum of compound **2** shows in the range from *δ* 1.78 to 4.23 ppm a different set of signals where the symmetric structure elements of a six-membered ring are now lost because of the skeletal change imposed by the pyrrolidine ring.

The characterization of compound **2** as impurity of Rapamycin (**1**), as required by regulatory institutions for the commercialization of APIs, has been conducted offering a complete spectroscopic pattern suitable for future investigation in the field of Rapamycin derivatives. Concerning the biological activity of compound **2** some indications and data are reported in the cited literature [1, 7]; in particular the mixed lymphocyte reaction inhibitory activity of Prolylrapamycin (**2**) was compared to that of Rapamycin (**1**) and found to decrease tenfold to demethylrapamycin analogs [7].

## Experimental section

All melting points (m.p.) are uncorrected. HRMS were done on a X500D QTOF system (ABSciex) available at the CGS of the University of Pavia. ^1^H, ^13^C and ^15^N NMR spectra were recorded in DMSO-*d*_6_ on a Bruker AV NEO 700 MHz spectrometer, equipped with a triple resonance helium cooled cryoprobe. Chemical shifts are expressed in ppm from the solvent residual peak (*δ*) and coupling constants (J) are in Hertz (Hz): *b*, broad; *s*, singlet; bs, broad singlet; *d*, doublet; *t*, triplet; *m*, multiplet. IR spectra (nujol mulls) were recorded on a spectrophotometer available at the Department and absorptions (ν) are in cm^−1^. Chromatographic details are reported in the starting and reference material section reporting the corresponding relative retention times (r.r.t.).

### Starting and reference materials

No authentic sample of the reported Prolylrapamycin **2** was available as a marker for its structure confirmation. As part of the study, the HPLC method used for the analysis of Rapamycin **1** was also used to evaluate the isolation and the purity of the peak at r.r.t 0.71, supposed to be the Prolylrapamycin **2**. The analyses of Rapamycin **1** were conducted by analytical HPLC on a Thermo BDS Hypersil column C18, 4.6 × 100 mm, 3 µm, with a mobile phase consisting of acetonitrile/ammonium acetate buffer 50/50 (v/v). The Rapamycin **1** injection sample was eluted using an isocratic run. A flow rate of 1.0 ml min^−^^1^ and UV detection at 278 nm were used.

Structural elucidation of impurities in drug substances is per se an improvement, with favorable impact both on knowledge and on the state of control of pharmaceutical processes. In this specific case, the clearly achieved attribution of Prolylrapamycin structure definitely confirmed our previous understanding that this typical impurity of Sirolimus manufacturing process is not originated by degradation of the drug substance. It is a process-related impurity. More specifically, a fermentation byproduct. A final assessment about this feature is essential from regulatory perspective as well (e.g. ICH Q3, Classification of Impurities).

Prolylrapamycin **2** was achieved via isolation of a small quantity (ca. 10–15 mg) of that impurity from the fermentation broth in the plant. A sample from the fermentation plant was taken for the laboratory trials. Fermentation broth for Rapamycin **1** contained around 2% (Area % in HPLC analysis) of an impurity having r.r.t. 0.71. The sample was enriched in that impurity initially by treatment on silica gel flash chromatography with eluent the mixture EtOAc/n-heptane in gradient proportion from 7/3 to 9/1. In this way a sample containing around 15% (Area % in HPLC analysis) of the impurity at r.r.t. 0.71 has been obtained (ca 1.0 g).

A Shimadzu preparative HPLC equipped with LC-20AP pumps, CBM-20A System controller, SPD20A UV–Vis detector, FRC10A Fraction collector and SIL10AP injector was used for preparative isolation of the impurity Prolylrapamycin **2** (r.r.t. 0.71). The injector was equipped with 5.0 ml sample loop and used in manual injection. Symmetry Prep C18, 30 × 100 mm, 5 µm particle size (Waters Inc, USA) was employed for isolation of Prolylrapamycin **2**. The flow rate was set at 20 ml min^−1^ and detection was carried out at 278 nm. Mobile phase consisting of A water and B acetonitrile was used. The following linear gradient was used: 0–1 min 50% B; 1–11 min increase from 50% B to 100% B; 11–20 min elution with 100% B; return to 50% B. Fractions containing Prolylrapamycin **2** were pooled and concentrated under reduced pressure on a Heidolph Rotavapor Model Laborota4000 to remove acetonitrile/water affording 20 mg of Prolylrapamycin **2**.

### Characterization of compound **1**

A sample of Sirolimus **1** (6.6 mg, 7.22 mmol) was dissolved in 0.5 ml DMSO-*d*_6_. 1D and 2D NMR spectra were collected at a 700 MHz NMR instrument at 298 K. For a complete characterization of the structure of compound **1** FT-IR spectra were collected as well as HRMS spectra and DSC analysis gave indication of the meting point of the product and relative decomposition upon heating.

**Compound 1**: straw yellows crystals. DSC analysis: mp 189.68 °C followed by endo peak due to immediate decomposition below 202.80 °C. IR: ν_OH_ 3412, ν_C=O_ 1716, ν_C=C_ 1644 cm^−1^. ^1^H NMR (DMSO-*d*_6_) *δ*: 0.60 and 1.91 (m, 1H + 1H, CH_2_); 0.74 (d, 3H, J = 7 Hz, CH_3_); 0.78 (d, 3H, J = 7 Hz, CH_3_); 0.83 (d, 3H, J = 7 Hz, CH_3_); 0.87 (d, 3H, J = 7 Hz, CH_3_); 0.97 and 1.06 (m, 1H + 1H, CH_2_); 0.99 (d, 3H, J = 7 Hz, CH_3_); 1.04 and 1.41 (m, 1H + 1H, CH_2_); 1.25 (m, 1H, CH); 0.86 and 1.53 (m, 1H + 1H, CH_2_); 1.18 and 1.75 (m, 1H + 1H, CH_2_); 1.19 and 1.83 (m, 1H + 1H, CH_2_); 1.26 and 1.86 (m, 1H + 1H, CH_2_); 1.41 and 1.66 (m, 1H + 1H, CH_2_); 1.28 and 1.59 (m, 1H + 1H, CH_2_); 1.53 (m, 2H, CH_2_); 1.59 and 2.11 (m, 1H + 1H, CH_2_); 1.64 (s, 3H, CH_3_); 1.68 (m, 1H, CH); 1.75 (s, 3H, CH_3_); 2.04 (m, 1H, CH); 2.23 (m, 1H, CH); 2.37 and 2.74 (m, 1H + 1H, CH_2_); 2.42 (m, 1H, CH); 2.83 (m, 1H, CH); 3.06 (s, 3H, OCH_3_); 3.16 (s, 3H, OCH_3_); 3.18 (m, 1H, CH); 3.18 and 3.44 (m, 1H + 1H, CH_2_); 3.27 (m, 1H, CH); 3.33 (s, 3H, OCH_3_); 3.64 (dd, 1H, J = 12, 2 Hz, CH); 3.96 (d, 1H, J = 5 Hz, CH); 4.01 (m, 1H, CH); 4.02 (m, 1H, CH); 4.60 (m, 1H, OH); 4.94 (d, 1H, J = 7 Hz, CH); 4.99 (m, 1H, CH); 5.09 (d, 1H, J = 10 Hz, CH=); 5.27 (m, 1H, OH); 5.47 (dd, 1H, J = 15, 9 Hz, CH=); 6.12 (dd, 1H, J = 11, 2 Hz, CH=); 6.14 (dd, 1H, J = 16, 11 Hz, CH=); 6.23 (dd, 1H, J = 15, 11 Hz, CH=); 6.41 (s, 1H, CH=); 6.47 (s, 1H, OH). ^13^C NMR (DMSO-*d*_6_) *δ*: 10.96; 13.85; 13.94; 16.03; 16.08; 15.21; 20.86; 22.14; 24.95; 26.72; 26.92; 30.11; 31.61; 32.99; 33.38; 33.85; 35.29; 35.69; 35.93; 38.88; 39.98; 40.06; 40.32; 40.52; 43.99; 45.70; 51.25; 55.96; 57.25; 57.42; 66.50; 73.71; 74.02; 76.23; 82.72; 84.22; 85.98; 99.51; 125.39; 127.49; 127.54; 130.93; 132.81; 137.63; 138.35; 139.79; 167.48; 169.70; 199.39; 208.05; 210.99. HRMS for C_51_H_79_NO_13_: calcd. 913.5551. Found: 913.5511.

### Characterization of compound **2**

A sample of Prolylrapamycin **2** (3.2 mg, 3.55 mmol) was dissolved in 0.5 ml DMSO-*d*_6_. 1D and 2D NMR spectra were collected at a 700 MHz NMR instrument at 298 K. For a complete characterization of the structure of compound **2** FT-IR spectra were collected as well as HRMS spectra and DSC analysis gave indication of the meting point of the product and relative decomposition upon heating.

**Compound 2**: straw yellows crystals. DSC analysis: large band starting from 123 °C and ending after 200 °C, centered at mp 161.14 °C; decomposition follows the melting with change of colour of the sample. IR: ν_OH_ 3404, ν_C=O_ 1716, ν_C=C_ 1634 cm^−1^. ^1^H NMR (DMSO-*d*_6_) *δ*: 0.61 and 1.87 (m, 1H + 1H, CH_2_); 0.71 (d, 3H, J = 7 Hz, CH_3_); 0.77 (d, 3H, J = 7 Hz, CH_3_); 0.81 (d, 3H, J = 7 Hz, CH_3_); 0.83 (d, 3H, J = 7 Hz, CH_3_); 0.70 and 1.54 (m, 1H + 1H, CH_2_); 0.99 (d, 3H, J = 7 Hz, CH_3_); 1.00 and 1.40 (m, 1H + 1H, CH_2_); 1.24 (m, 1H, CH); 0.95 and 1.12 (m, 1H + 1H, CH_2_); 1.16 and 1.74 (m, 1H + 1H, CH_2_); 1.15 and 1.86 (m, 1H + 1H, CH_2_); 1.17 and 1.89 (m, 1H + 1H, CH_2_); 1.78 and 1.81 (m, 1H + 1H, CH_2_); 1.90 and 2.22 (m, 1H + 1H, CH_2_); 1.58 (m, 2H, CH_2_); 1.62 (s, 3H, CH_3_); 1.76 (m, 1H, CH); 1.78 (s, 3H, CH_3_); 2.05 (m, 1H, CH); 2.24 (m, 1H, CH); 2.25 (m, 1H, CH); 2.29 and 2.72 (m, 1H + 1H, CH_2_); 2.81 (m, 1H, CH); 3.07 (s, 3H, OCH_3_); 3.16 (m, 1H, CH); 3.18 (s, 3H, OCH_3_); 3.18 (m, 1H, CH); 3.23 (m, 1H, CH); 3.24 and 3.56 (m, 1H + 1H, CH_2_); 3.31 (s, 3H, OCH_3_); 3.67 (dd, 1H, J = 11, 2 Hz, CH); 4.03 (m, 1H, CH); 4.05 (m, 1H, CH); 4.18 (d, 1H, J = 3 Hz, CH); 4.23 (dd, 1H, J = 8, 4 Hz, CH); 4.59 (m, 1H, OH); 4.88 (m, 1H, CH); 5.12 (d, 1H, J = 10 Hz, CH=); 5.25 (m, 1H, OH); 5.47 (dd, 1H, J = 15, 10 Hz, CH = ); 6.14 (dd, 1H, J = 15, 11 Hz, CH=); 6.22 (d, 1H, J = 15 Hz, CH=); 6.29 (dd, 1H, J = 15, 11 Hz, CH=); 6.43 (dd, 1H, J = 15, 11 Hz, CH=); 6.50 (s, 1H, OH). ^13^C NMR (DMSO-*d*_6_) *δ*: 10.77; 12.71; 14.88; 15.43; 15.98; 16.11; 22.01; 24.54; 26.44; 29.40; 29.92; 31.37; 33.00; 33.06; 33.47; 34.69; 35.66; 35.95; 39.23; 39.79; 40.49; 40.85; 41.27; 44.98; 47.50; 55.98; 57.07; 57.24; 59.00; 66.34; 73.62; 73.75; 76.33; 82.43; 84.26; 85.16; 99.75; 124.43; 127.61; 127.71; 131.04; 132.95; 137.01; 138.21; 139.58; 166.03; 170.92; 198.66; 208.20; 210.03. HRMS for C_50_H_77_NO_13_: calcd. 899.5395. Found: 899.5336.

### Supplementary information

Supplemental material
