# Supplementary material for: Structure of prolylrapamycin: confirmation through a revised and detailed NMR assignment study
Source: J Antibiot (Tokyo). 2024 Mar 19;77(6):345–52. doi: 10.1038/s41429-024-00714-6 (PMC11128375; doi:10.1038/s41429-024-00714-6)
Supplement: Supplementary file 1 — Supplemental material [file 41429_2024_714_MOESM1_ESM.docx]

**SUPPLEMENTAL MATERIAL**

Structure of Prolylrapamycin: Confirmation through a Revised and Detailed NMR Assignment Study

Annalisa Mortoni,^a*^ Eugenio Castelli,^a^ Teresa Recca,^b^ Paolo Quadrelli^c^*

a. Curia Italy S.r.l., Via Volturno 43, 20089 Rozzano (MI), Italy

b. Centro Grandi Strumenti (CGS), Università degli Studi di Pavia, Via Bassi 21, 27100 – Pavia, Italy

c. Dipartimento di Chimica, Università degli Studi di Pavia, Viale Taramelli 12, 27100 – Pavia, Italy

**CONTENT**

1. NMR spectra of compound **1** Pag. S2
2. NMR spectra of compound **2** Pag. S7
3. DSC analyses Pag. S11

1. NMR spectra of compound **1**

^15^N-HMBC


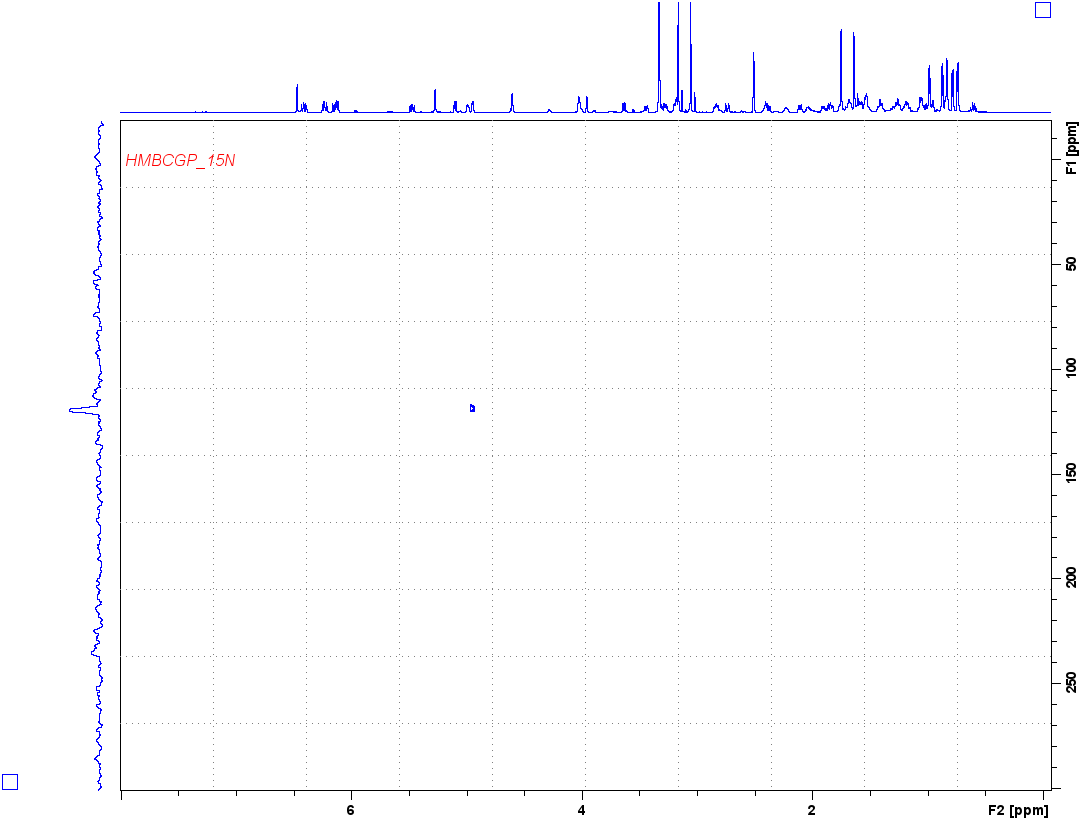


COSY


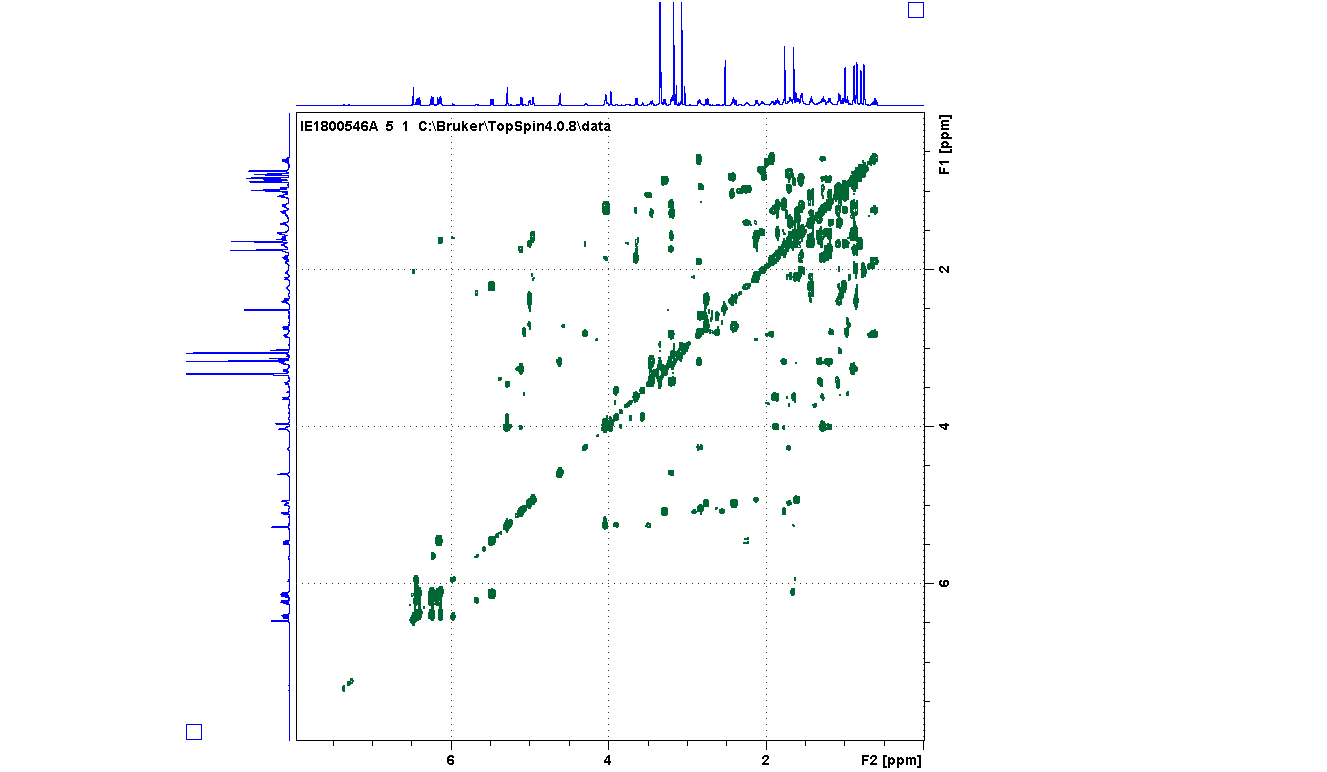


HMBC


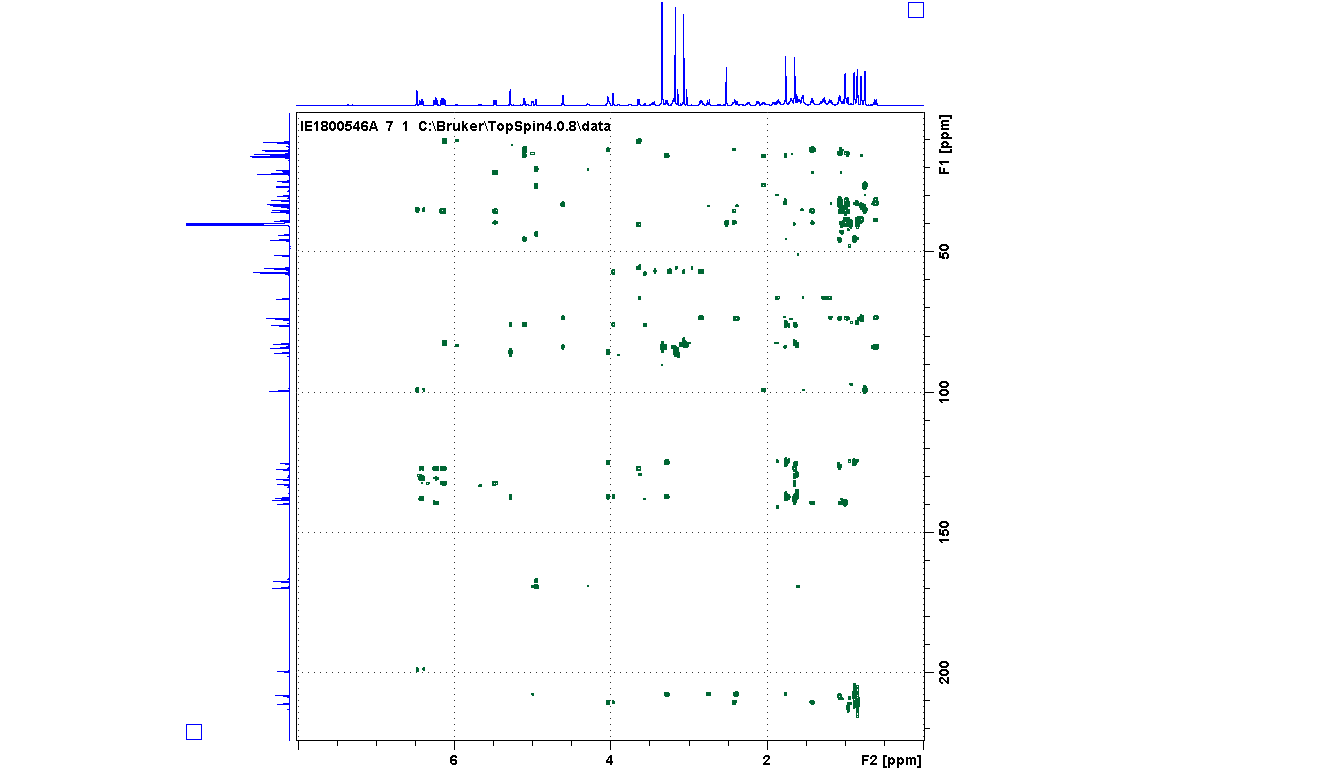


HSQC


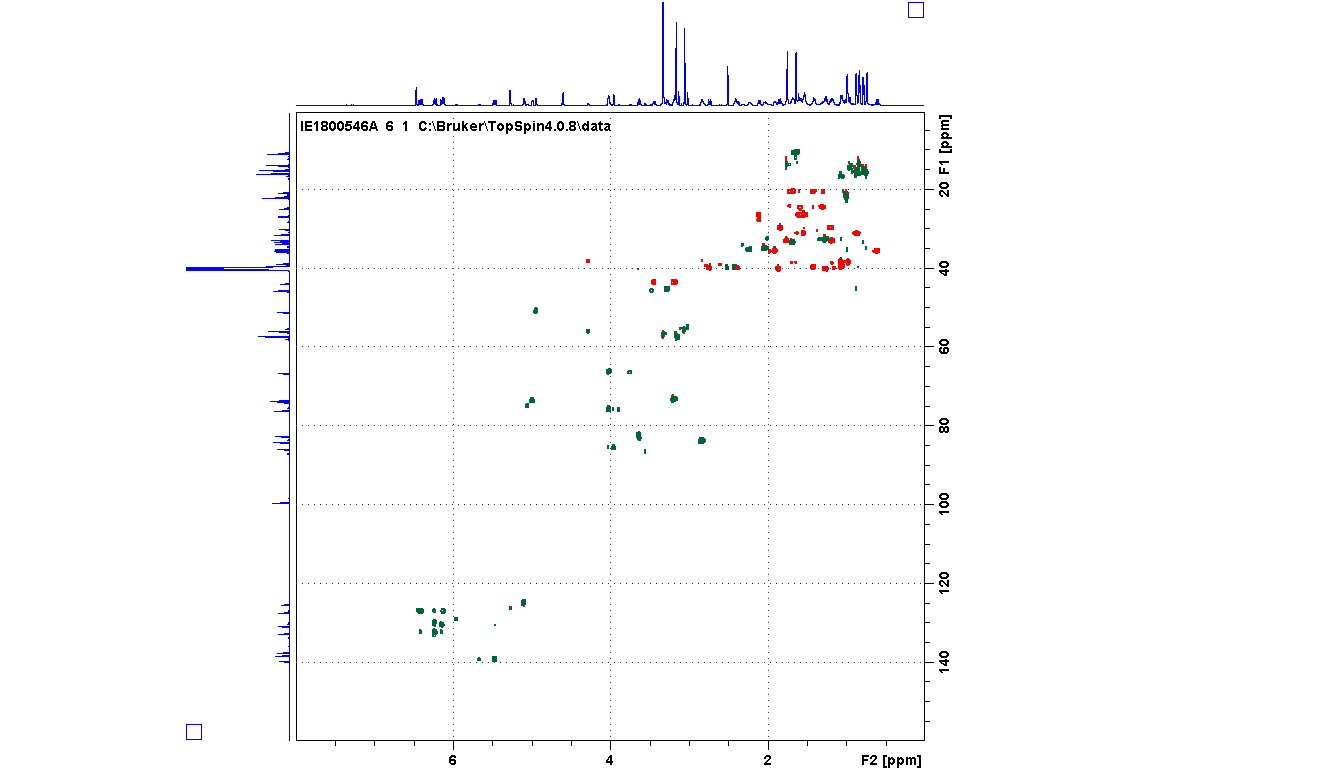


NOESY


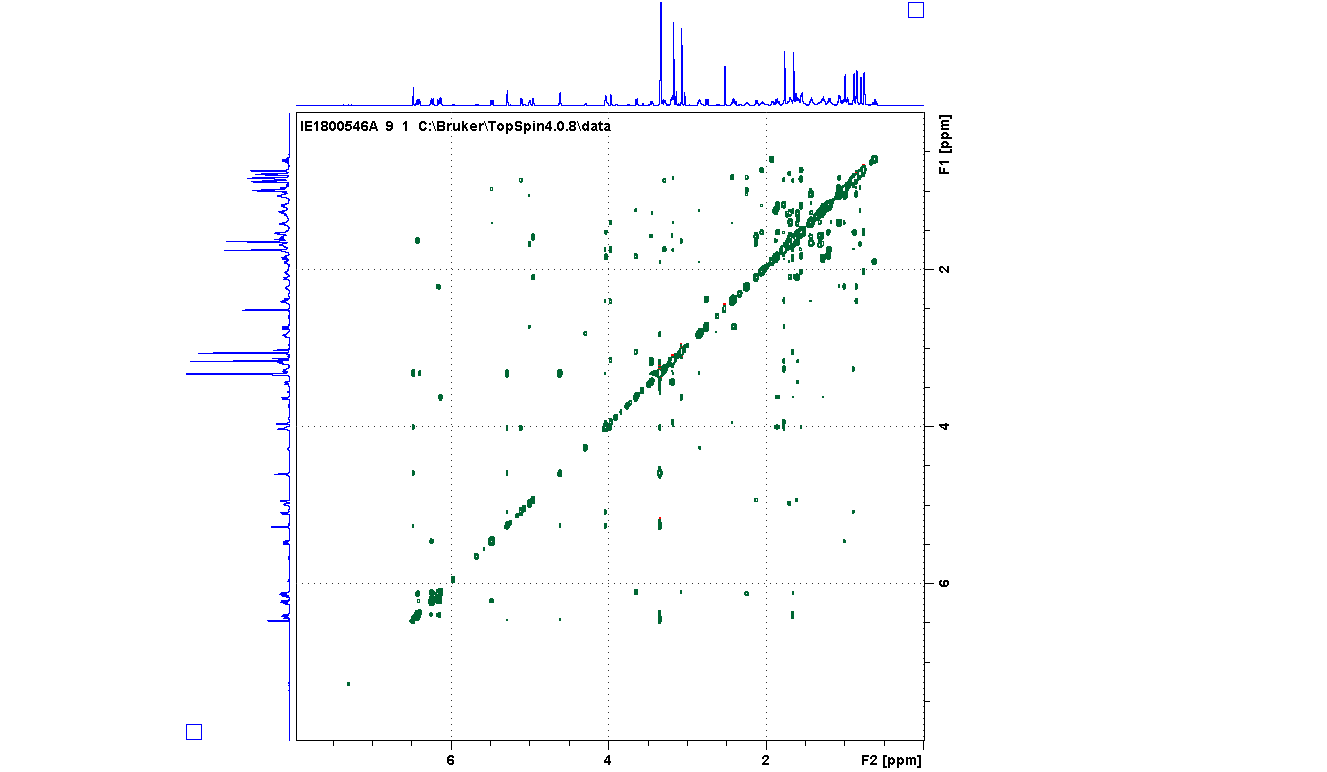


TOCSY


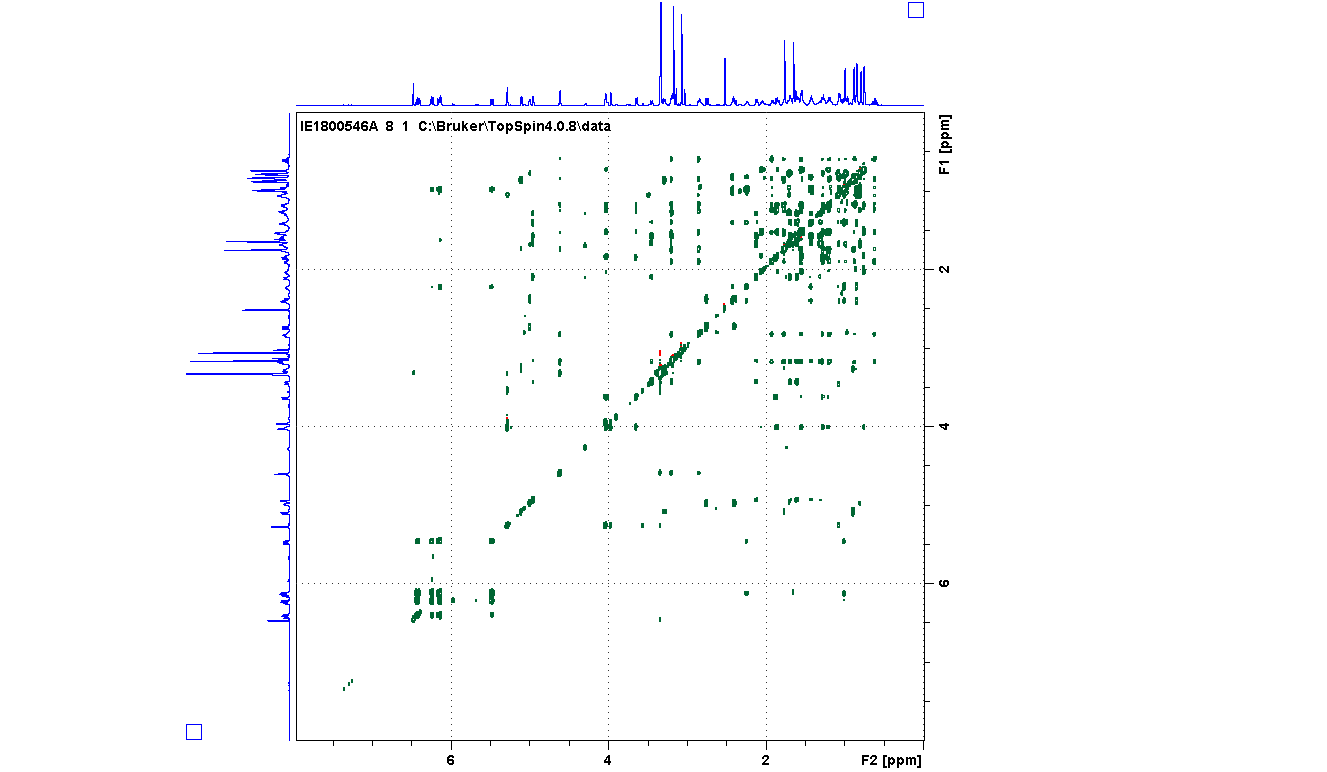


2. NMR spectra of compound **2**

^15^N-HMBC


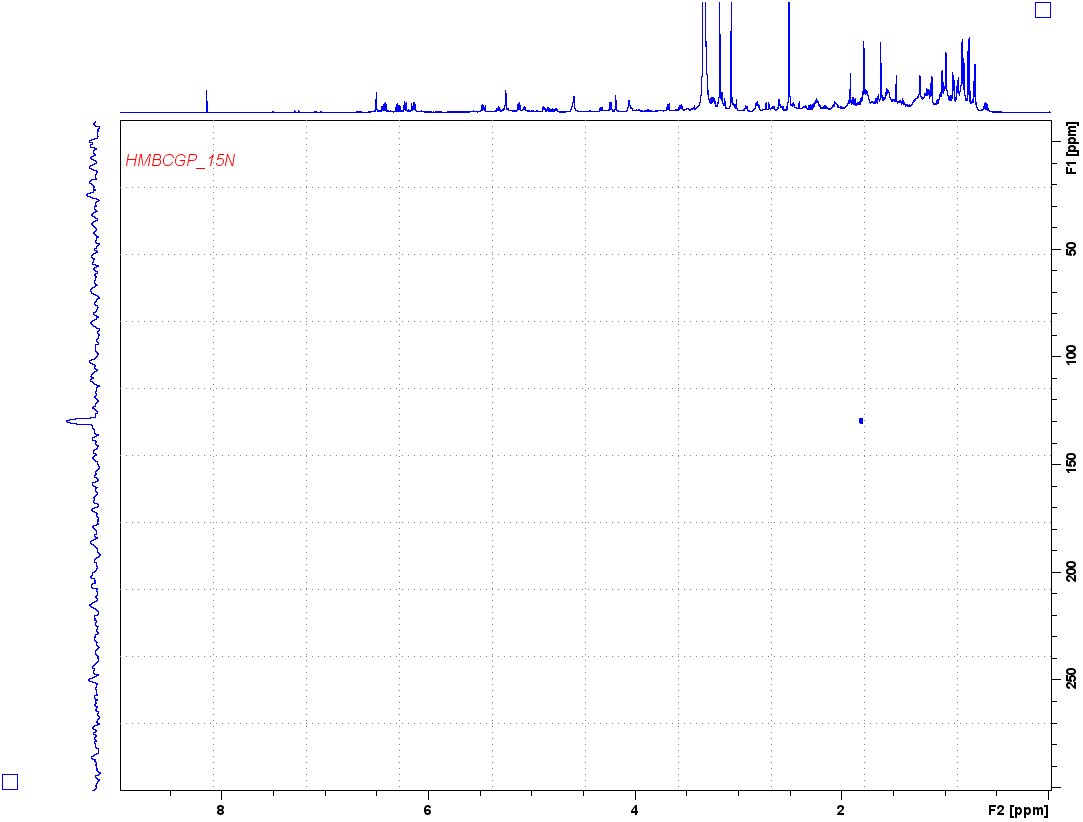


COSY


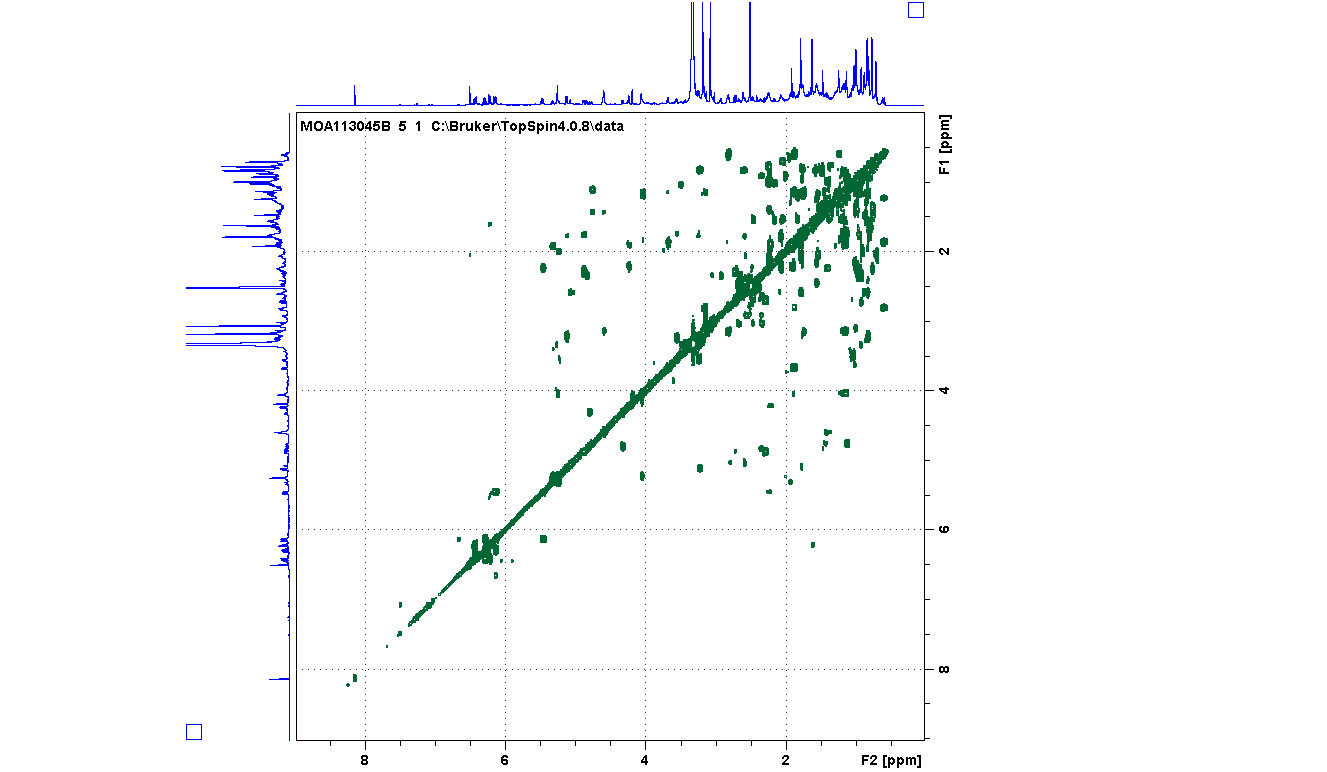


HMBC


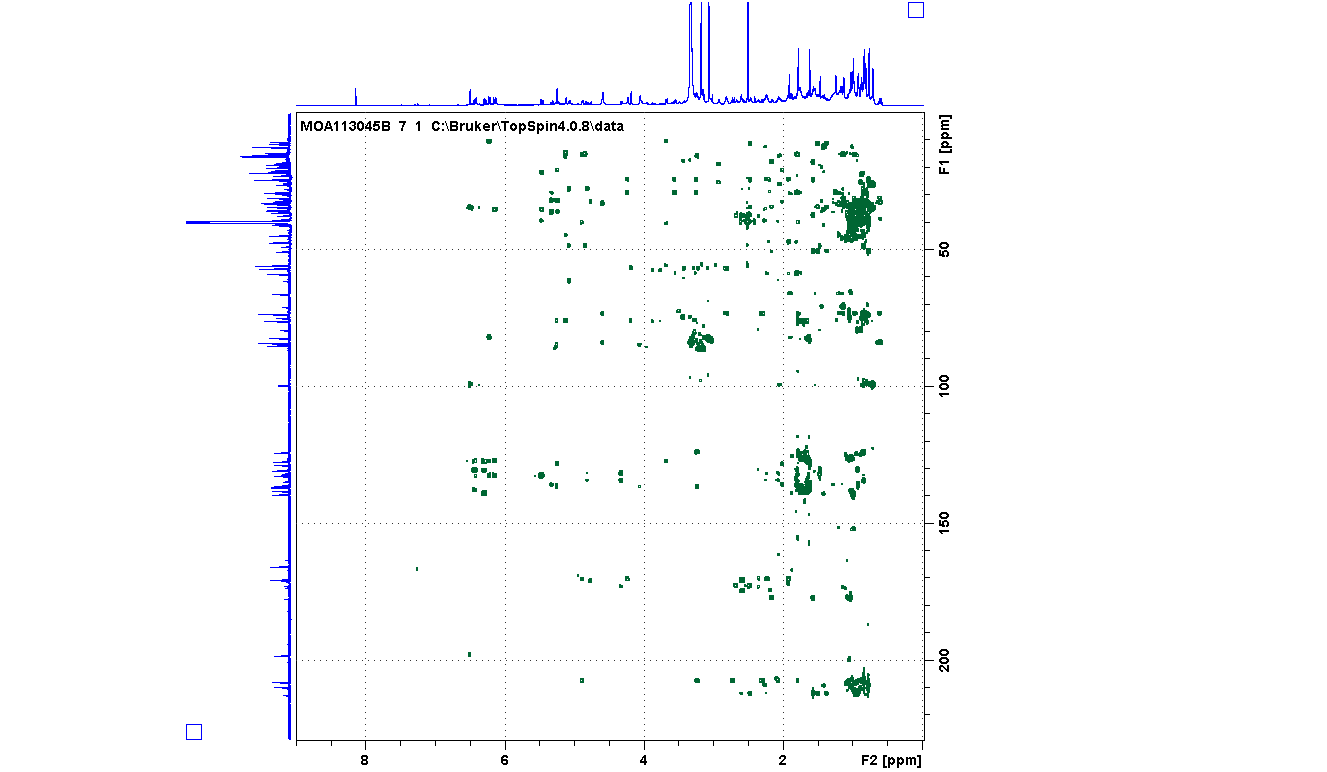


HSQC


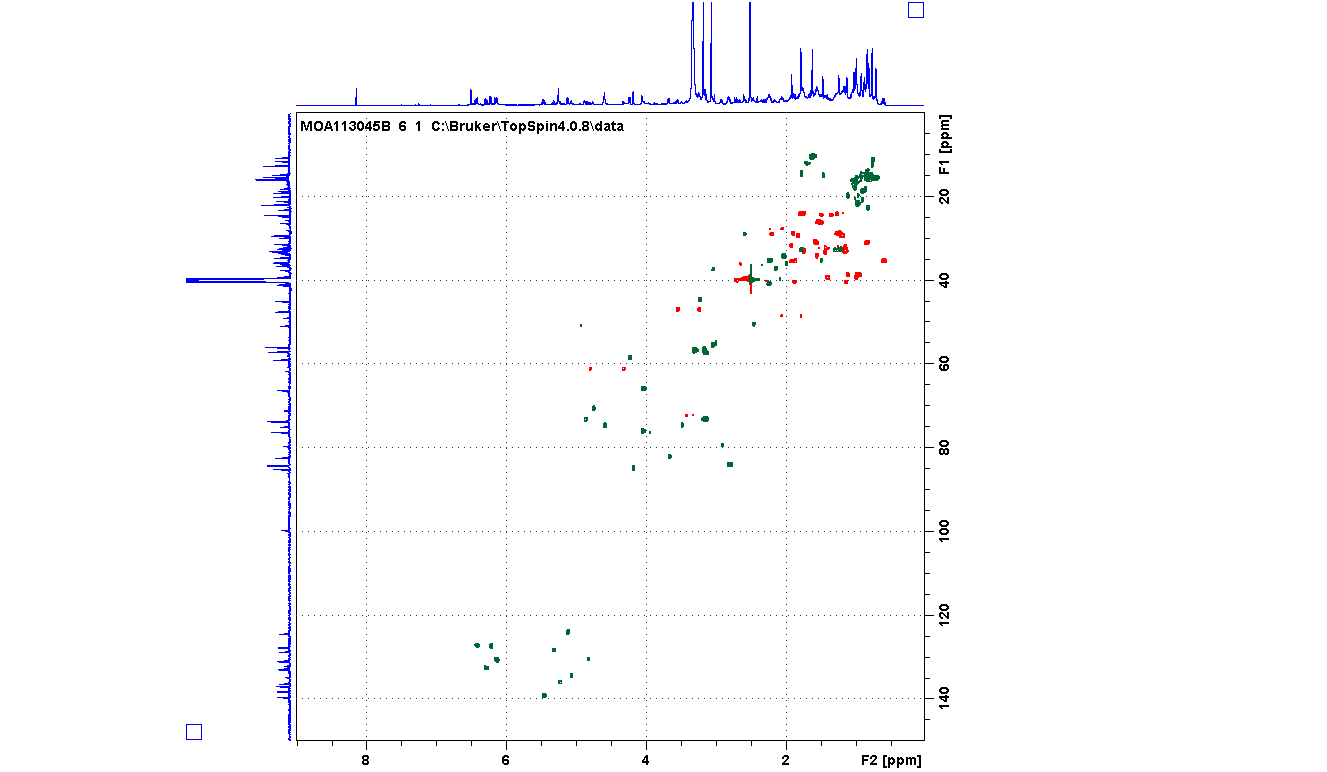


NOESY


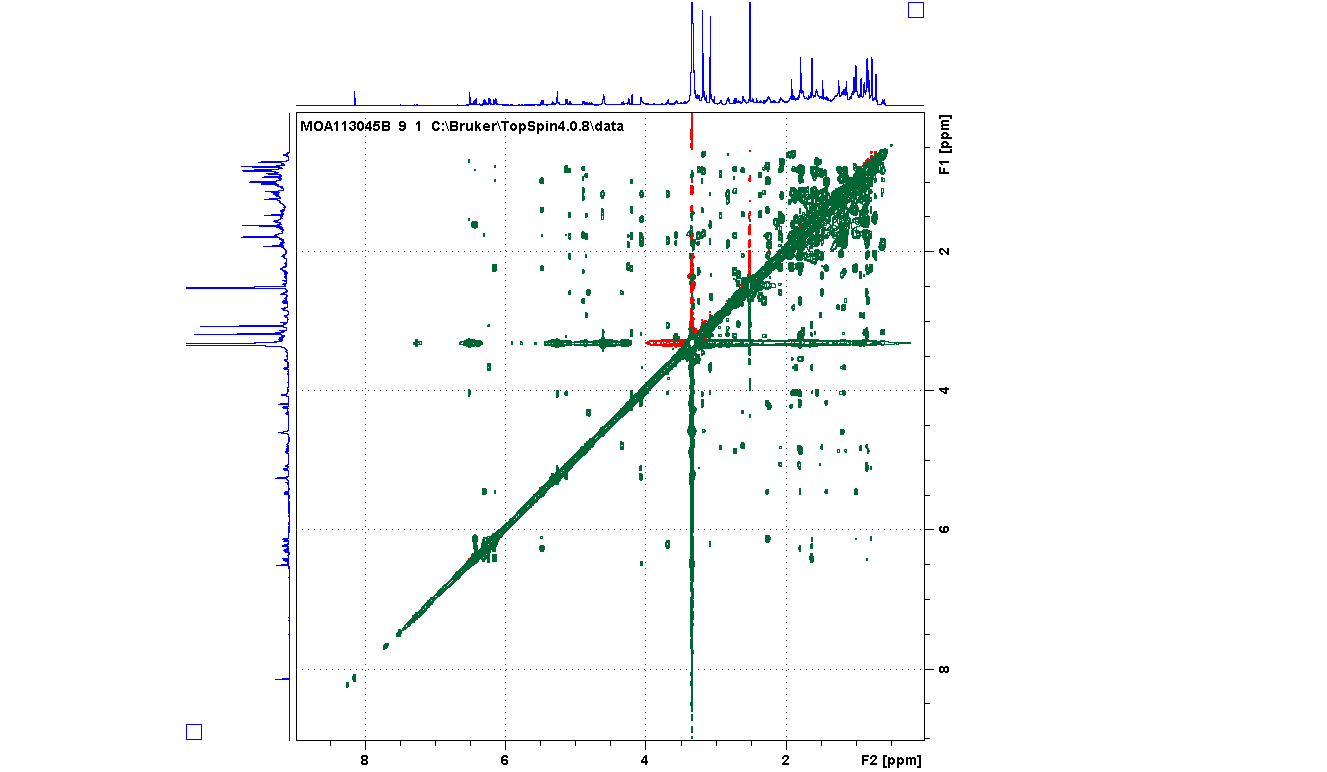


TOCSY


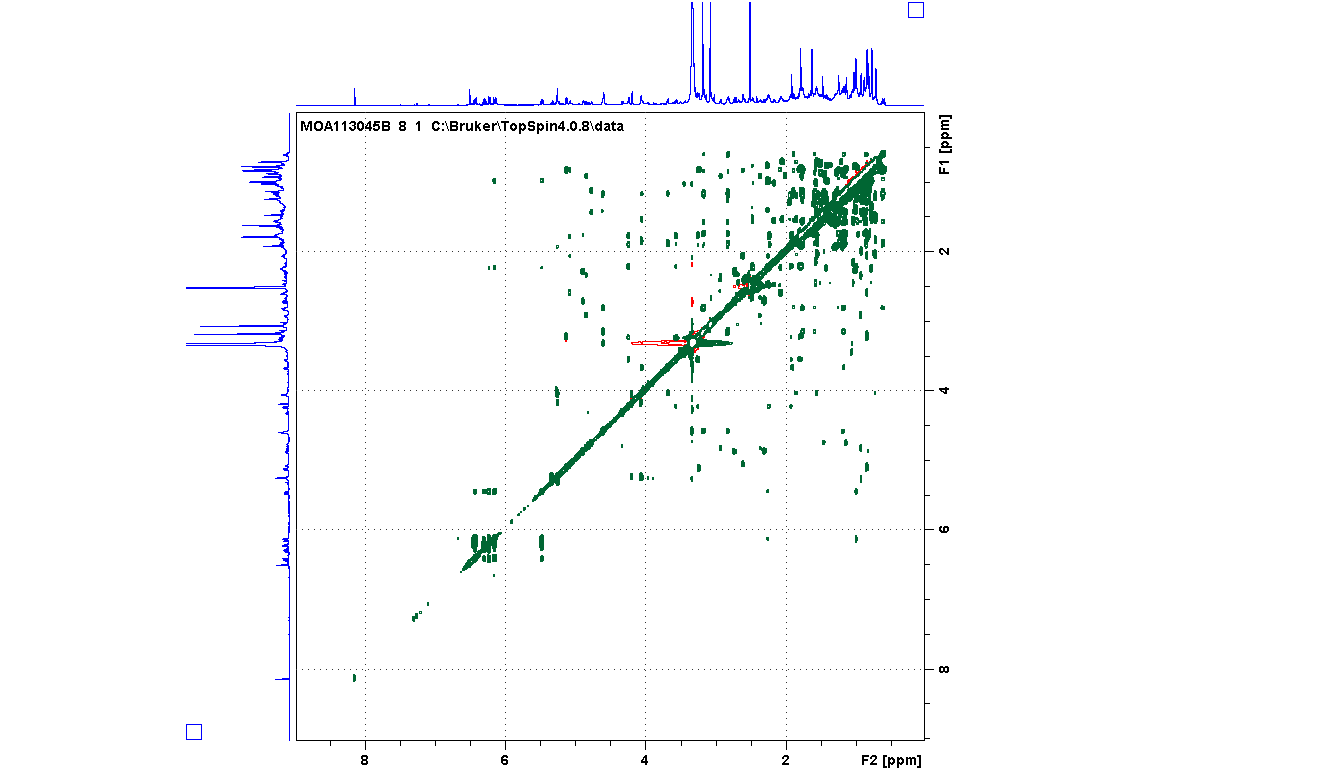


3. DSC analyses

Compound **1**

Compound **2**
